# Recent Advances in the Development of Tetrazine Ligation Tools for Pretargeted Nuclear Imaging

**DOI:** 10.3390/ph15060685

**Published:** 2022-05-30

**Authors:** Rocío García-Vázquez, Umberto Maria Battisti, Matthias M. Herth

**Affiliations:** 1 Department of Drug Design and Pharmacology, Faculty of Health and Medical Sciences, University of Copenhagen, Universitetsparken 2, 2100 Copenhagen, Denmark; rociogv@sund.ku.dk (R.G.-V.); umberto.battisti@sund.ku.dk (U.M.B.); 2 Department of Clinical Physiology, Nuclear Medicine & PET, Rigshospitalet, Blegdamsvej 9, 2100 Copenhagen, Denmark

**Keywords:** tetrazine ligation, bio-orthogonal chemistry, pretargeted imaging, molecular imaging

## Abstract

Tetrazine ligation has gained interest as a bio-orthogonal chemistry tool within the last decade. In nuclear medicine, tetrazine ligation is currently being explored for pretargeted approaches, which have the potential to revolutionize state-of-the-art theranostic strategies. Pretargeting has been shown to increase target-to-background ratios for radiopharmaceuticals based on nanomedicines, especially within early timeframes. This allows the use of radionuclides with short half-lives which are more suited for clinical applications. Pretargeting bears the potential to increase the therapeutic dose delivered to the target as well as reduce the respective dose to healthy tissue. Combined with the possibility to be applied for diagnostic imaging, pretargeting could be optimal for theranostic approaches. In this review, we highlight efforts that have been made to radiolabel tetrazines with an emphasis on imaging.

## 1. Introduction

**Figure 1:**
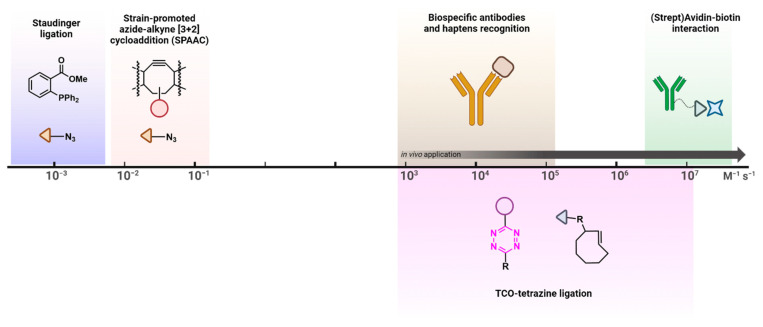
Comparison of state-of-the-art bio-orthogonal reactions with respect to their kinetics. Rate constants (k2) are reported. Reported values are measured in aqueous solution at a 1 µM concentration of both probes.

Bio-orthogonal reactions are transformations that can occur in living organisms without interfering with any biochemical processes [1,2,3,4]. They have been applied, for example, for pretargeting (Figure 1) [2,5,6,7]. Pretargeting can, for instance, be used to increase imaging contrast of nanomedicines, reduce radiation doses to healthy tissue, or trigger drug release [6,8,9]. Several bio-orthogonal reactions have been described over the years, each with specific advantages as well as limitations [10,11,12]. Most reactions found applications in vitro; however, only a few could successfully be applied in a real in vivo setting [3,13]. The first bio-orthogonal reaction, the Staudinger ligation, was developed in 2000 by Saxon and Bertozzi [14]. Shortly after, the strain-promoted alkyne–azide cycloaddition (SPAAC) was described and successfully applied in 2004 [4,15]. Unfortunately, both reactions have been shown to be difficult to translate to in vivo experiments in mammals. Necessary concentrations required for the relatively low-rate constants of these reactions made them only compatible in very few applications [14,15,16,17,18]. Especially for nuclear molecular imaging applications, where tracer amounts (nmol) are typically used, the required concentrations of the Staudinger ligation or the SPAAC are typically not reachable. In 2008, Fox et al. proposed the tetrazine ligation between an electron-deficient tetrazine (Tz) and a strained trans-cyclooctene (TCO) derivative as a new bio-orthogonal reaction [19,20]. High specificity, inertness to biological media, and impressive rate constant of up to 10^7^ M^−1^ s^−1^ compared to other bio-orthogonal reactions make the tetrazine ligation the perfect tool for in vivo applications [21]. In comparison to clinically applied pretargeting pairs, i.e., the bispecific antibody and hapten recognition as well as the (strep)avidin–biotin interaction, rate constants are comparable between these ligations. However, Tz ligation results in covalent bond formation and is, therefore, completely irreversible. In contrast, the bispecific antibody and hapten recognition, as well as the (strep)avidin–biotin interaction, is driven by noncovalent high affinity interactions, which make them partly reversible over time in vivo [18]. Another advantage of Tz ligation is that the reaction is based on small molecules, which can be more easily upscaled, have their rate constants manipulated, and have their physiochemical properties designed for specific applications—for example, to enter the brain [18,22]. Tz ligation can also be used for “click-to-release” strategies that have lately been proven to be more effective than their conventional counterparts. Bio-orthogonally triggered drug release increased median survival from 26 days to 50 days in rodents [23]. Clinical Phase I studies were initiated in 2020 [24]. This adds a completely new dimension to the use of Tz ligation for bio-orthogonal applications.

For these reasons, we will focus this review on Tz ligation with respect to its chemical basis and its application as a pretargeting nuclear imaging tool. We will also discuss recent molecular insights on this reaction and review the latest labeling advances. Pretargeted radiotherapy, or “click-to-release”, strategies are beyond the scope of this work and can be reviewed elsewhere [18,25,26]. 

## 2. The Tetrazine–TCO Ligation

**Figure 2:**
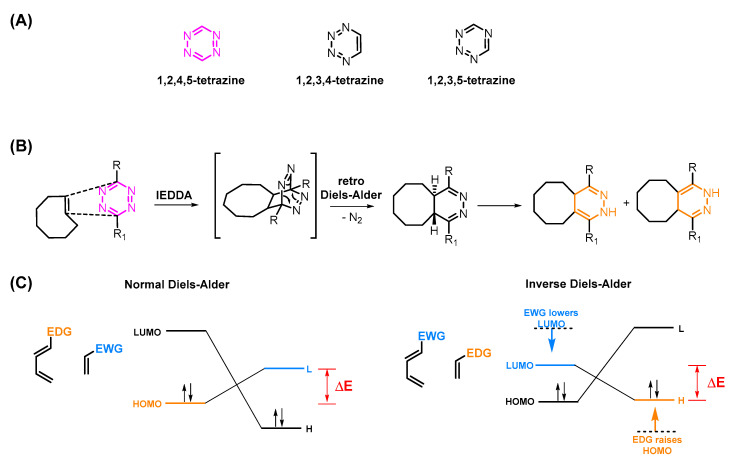
(**A**) Tetrazine isomers. (**B**) Mechanism of the Tz ligation, consisting of an inverse electron-demand Diels–Alder[4 + 2] cycloaddition (IEDDA) between a tetrazine (Tz) and a trans-cyclooctene (TCO) derivative, followed by a retro-Diels–Alder reaction (retro-DA) and N_2_ elimination. (**C**) Frontier orbital model of the Diels–Alder reaction and the IEDDA reaction. EDG: electron donating group, EWG: electron withdrawing group, HOMO: highest occupied molecular orbital, LUMO: lowest unoccupied molecular orbital. By electronically tuning IEDDA derivatives, it is possible to manipulate the LUMO_Diene_–HOMO_Dienophile_ energy gap (ΔE) and accelerate the IEDDA. For the diene pair, electron deficiency derivatives decrease the energy of the LUMO_Diene_; as for the dienophile, electron-rich derivatives increase the energy of the HOMO_Dienophile_.

Tetrazines consist of a six-membered aromatic ring containing four nitrogens (Figure 2A) [27,28]. Among three different possible isomers, 1,2,4,5-tetrazines are the only structures used for Tz ligation [29]. This reaction is initiated via an inverse electron-demand Diels–Alder [4 + 2] cycloaddition (IEDDA) and followed by a retro-Diels–Alder reaction (retro-DA) (Figure 2B). In contrast to the standard Diels–Alder reaction (DA), the initial IEDDA of the Tz ligation is characterized by diene/dienophile pairs with an opposite electronic character, i.e., an electron-deficient diene (Tz) reacts with an electron-rich dienophile (most often a TCO). Consequently, the lowest unoccupied molecular orbital (LUMO_Diene_) of the Tz reacts with the highest occupied molecular orbital (HOMO_Dienophile_) of the TCO (Figure 2C) [30]. This IEDDA is the rate-determining step of the ligation and can be influenced by reducing the energy gap between the HOMO_Dienophile_ and the LUMO_Diene_. This can, for example, be achieved by lowering the electron density of the Tz or increasing that of the TCO. Several studies have been published aiming to increase reactivity [13,22,31,32]. As mentioned before, the IEDDA is followed by a retro-DA, in which nitrogen gas is eliminated to form either dihydropyridazine or pyridazine adducts (Figure 2B). A deeper mechanistic review was published by Oliveira et al. [13,31,33,34].

## 3. Influencing the Reaction Kinetics of the Tetrazine Ligation

The reaction kinetics of the Tz ligation are dependent on the electronic characters of the Tz and the TCO. In general, the higher the electron density of the TCO and the lower the electron density of the Tz, the faster the reaction. 

### 3.1. Reactivity of TCOs

**Figure 3:**
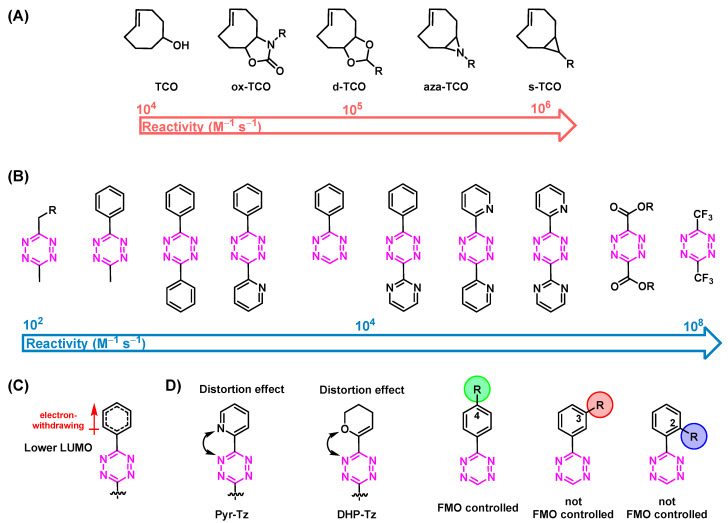
(**A**) Structures and reactivity order of TCOs. Increased strain leads to higher reactivity. Reactivity calculated with bispyridyl Tz, as reported in ref. [39]. (**B**) Structures and reactivity order of Tz scaffolds. The activity is dependent on electronic and steric effects. (**C**) Tz can be activated by conjugation of an electron-withdrawing group (EWG) to the aromatic Tz system. The LUMO_Diene_ will be shifted to a lower energy level. (**D**) The substitution pattern also influences the Tz’s reactivity. Distortion, steric effects, and solvent interactions are responsible for the observed reactivity trend.

The reactivity of TCOs can be increased by conjugating electron donating groups (EDGs) to the TCO moiety. This raises both the HOMO and the TCO to a higher energy level and consequently decreases the energy gap between the highest occupied molecular orbital (HOMO_Dienophile_) of the TCO and the lowest unoccupied molecular orbital (LUMO_Diene_) of the Tz (Figure 2C). Another way to increase the reactivity of the diene is to increase the strain of the diene. In fact, this is the main reason why TCOs are so much more reactive than cis-cyclooctenes (CCOs) (reactivity increase by factor of 100,000) [19,35,36,37]. Additional ring strain further increases the reactivity (Figure 3A). However, the stability of the TCOs usually correlates with their reactivity, limiting the possibility of maximizing the latter, especially for in vivo applications. Therefore, most studies are performed with “standard” TCOs and not with ox-TCO, d-TCO, aza-TCO, or s-TCO, for example [38,39].

### 3.2. Reactivity of Tzs

Tetrazines can be further activated by conjugation of an electron-withdrawing group (EWG) to the aromatic Tz system. Consequently, the LUMO_Diene_ will be shifted to a lower energy level. This results in a reactivity increase, as displayed in Figure 3B [29,40,41,42]. The steric effects of Tz substituents also have a pivotal role. In general, the smaller the substituent, the more reactive the Tz. Steric effects are the main reasons why monosubstituted 3-phenyl-1,2,4,5-tetrazines (H-Tzs) typically have 70-fold-increased activity compared to methyl- or phenyl-substituted Tzs [43]. Recently, Svatunek and al. showed that distortion effects between the aromatic and the tetrazine rings can increase the reactivity of the molecule [44]. Increased distortion led to faster rate constants (Figure 3C) [44]. The addition of substituted phenyl rings to the Tz is frequently carried out, for example, to be able to label the Tz. The position of these substituents also have a strong influence on the reactivity of the Tz [22]. Whereas the reactivity of 4-phenyl substituted H-Tzs can be predicted solely based on the frontier molecular orbital theory (FMO), 2- and 3-phenyl-substituted H-Tzs do not follow these considerations. Other factors such as ring distortion, steric bulk of substituents, and solvent interaction must be taken into account to explain their reactivity [22]. Figure 3 displays typically employed Tz scaffolds. As is the case for TCOs, Tzs with increased reactivity show decreased stability. Reactivity levels between 10^−3^–10^−6^ appear to be the limit in vivo. Bispyridyl-based and H-Tz are the most employed Tzs in vivo due to their high-rate constants and relative stability [6,45,46]. 

## 4. The Use of Nanomedicines for Molecular Imaging

Nuclear medicine has become an important tool for early diagnosis and therapy of diseases in the fields of oncology, cardiology, and neurology [47]. Molecular imaging techniques such as positron emission tomography (PET) or single-photon emission computed tomography (SPECT), using adequate radiolabeled derivatives, allow the visualization of biological processes in living organisms [48,49,50]. These techniques are noninvasive, highly sensitive (the level of detection approaches 10^−12^ M of tracer), and offer isotropism (i.e., the ability to detect organ accumulation accurately, regardless of tissue depth) [51,52]. Compared to SPECT, PET enables a quantitative measure of the tracer delivered to the target. This is mainly related to the greater spatial and temporal resolution of clinical PET cameras, which are at least ten times more sensitive. For these reasons, PET images have better quality and contrast at lower radiation doses. The selection between PET and SPECT imaging depends on the properties of the radionuclides, the corresponding structure to which they adhere, the selected nuclear imaging approach, and the application of the radiotracer [53,54,55].

Radionuclides frequently used in SPECT and PET include fluorine-18 (110 min), gallium-68 (68 min), carbon-11 (20.4 min), and technetium-99 m (6.01 h), as they are considered short-half-life isotopes resulting in less radiation burden for healthy tissues [56,57]. However, it is important that the biological half-life of the target vector matches the half-life of the radionuclide; therefore, many other isotopes have been used for the radiolabeling of tracers with slower pharmacokinetic properties (Table 1) [18,58].

The use of nanomedicines for the development of radiotracers has increased recently, as they have great potential to improve both disease diagnosis and therapy [59,61]. A wide range of materials, such as polymers, monoclonal antibodies (mAbs), and liposomes, has been exploited [18,64,65]. The modes of action of nanomedicines differ from one another. However, one of the main disadvantages they have in common is their slow pharmacokinetics, which can be matched only with long-lived isotopes, e.g., indium-111, iodine-124, or zirconium-89 [66]. These radionuclides have half-lives long enough to image the in vivo behavior of nanomedicines over several days, which is generally the period of time required to obtain a good contrast between the target and the surrounding tissues [67,68,69]. However, this also results in an undesirable significant radiation dose for patients [70].

## 5. Pretargeted Nuclear Imaging 

**Figure 4:**
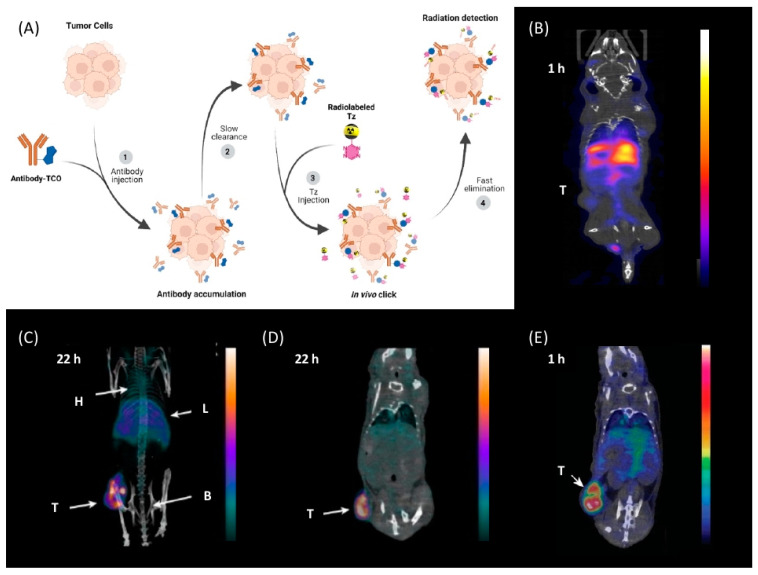
(**A**) Visualization of the pretargeting based on tetrazine. (**B**–**E**) Pretargeted imaging of CC49-TCO injected 72 h before the effector molecule in mice bearing LS174T tumor xenograft. (**B**) Conventional imaging of ^111^In-label CC49 after 1 h, low uptake is observed in the tumor; (**C**) SPECT/CT images with [^111^In]**1** 22 h after Tz imaging agent injection. High tumor uptake is observed. KGaA, Weinheim [6,75]. (**D**) PET/CT image with [^64^Cu]**4** in mice bearing the LS174T tumor xenograft. High tumor uptake is observed [75]. (**E**) PET/CT image with [^18^F]**25** in mice bearing the LS174T tumor xenograft. High tumor uptake is already observed one hour after injection. Reprinted/adapted with permission from [76]. Copyright © 2021, American Chemical Society.

Over the years, pretargeting imaging has emerged as a unique possibility to increase imaging contrast and reduce the undesirable radiation dose when using nanomedicines as targeting vectors for imaging or therapy [3,18,71,72]. In particular, tetrazine ligation has attracted immense attention for this purpose due to its peculiar properties. Pretargeting is based on a two-step strategy which allows the separation of the targeting process of nanomedicines from the actual imaging step. In this two-step method, a tagged nanomedicine, e.g., a mAb, is first administered and allowed to accumulate at its target and be excreted from the blood stream prior to the injection of a small molecule (effector molecule) that selectively binds to tag of the nanomedicine. This strategy makes use of the unique targeting properties of nanomedicines as well as the favorable pharmacokinetics of small molecules (Figure 4) [18,73]. Consequently, short-lived radionuclides with favorable radiophysical properties can be used [5,74]. In principle, the Tz or TCO fraction can be used as labels. Thus, it would be common to use the more stable derivative to modify the nanomedicine since it will be in circulation longer and the less stable molecule as the radioligand since it will be eliminated faster. The reactivity and stability of TCO derivatives have been investigated, showing that the stability of the metabolic fraction of TCO is stabilized by using shorter linkers to the nanomedicine. Biological half-lives of 6 days have been demonstrated with this approach [20]. Consequently, the use of Tzs as effector molecules has gradually emerged as a more practical approach for pretargeting [18].

## 6. Tetrazine Labeling

Attempts to radiolabel Tzs can be divided into two categories: (1) radiometal (Table 2) or (2) non-radiometal approaches (Table 3 and Table 4). Within this review, we followed the nomenclature suggested by Herth et al. in 2021 [77].

## 7. Radiometals

The vast majority of strategies to radiolabel Tzs have been based thus far on strategies using radiometals and chelators. This is not surprising, as labeling can be carried out under relatively mild conditions (e.g., no strong basic environment is needed). Pairing diagnostic with therapeutic radionuclides, enabling theranostic approaches, is a clear advantage of this strategy. Upscaling and distribution range are typical concerns. From a chemical point of view, radiometal labeling usually results in polar compounds, i.e., the resulting Tzs cannot reach intracellular targets or cross the blood–brain barrier (BBB). In the following, we will discuss current approaches and radionuclides used thus far. 

**Table 2:** Overview of radiometal labeled Tzs from 2011–2021 (list is not exhaustive).

NO.	Chemical Structure	Refs.	NO.	Chemical Structure	Refs.
**1**	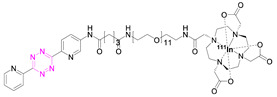	[6,75]	7	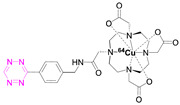	[78]
**2**	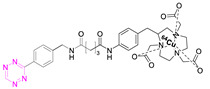	[79,80]	**8**	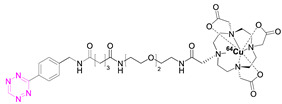	[81]
**3**	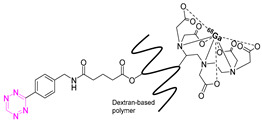	[82]	**9**	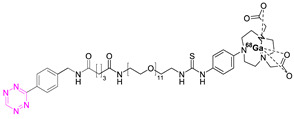	[45]
**4**	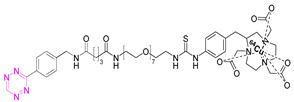	[75,80]	**10**	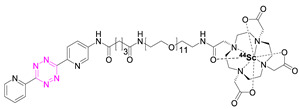	[83]
**5**	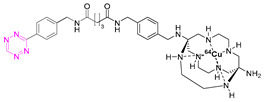	[80]	**11**	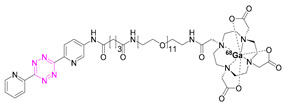	[84]
**6**	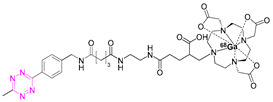	[85]			

### 7.1. Indium-111

The first successful pretargeted imaging study based on tetrazine ligation was already published by Rossin et al. in 2010. The effector molecule was based on an ^111^In-labeled Tz ([^111^In]**1**) [6]. The half-life of this SPECT radionuclide is 2.8 days and, as such, is not optimally suited for imaging purposes, as in vivo radiolabeled nanomedicines are radioactive for a relatively long time and non-target-bound radioactivity leads to an unnecessary radiation burden. However, the used respective effector molecule is still one of the best pretargeted imaging agents available to this day [75,86], and the study paved the road for the development of a completely new imaging approach [6]. The design of the study utilized TCO-tagged CC49, a non-internalizing, mAb-targeting, tumor-associated glycoprotein 72 (TAG-72). This antigen is highly expressed on human colon LS174T cancer cells, which were used to establish a xenograft bearing mice model. The non-internalizing mAb was chosen as [^111^In]**1** cannot penetrate through cell membranes. Consequently, the vector availability is maximized using a non-internalizing mAb. Pretargeting experiments were carried out by injecting CC49-TCO (100 µg, 7.4 TCOs/mAb) 24 h prior to [^111^In]**1** administration (21 µg, 3.4 equiv. to TCO, 20–50 MBq). Imaging was conducted 3 h post-injection (p.i.); 4.2% injected dose per gram (% ID/g) accumulated in the tumor and a tumor-to-muscle ratio of 13:1 were observed. Minimal tumor accumulation was detected in control experiments, where mice were injected with either CC49 or TCO-modified rituximab—a targeting vector that does not target TAG-72. 

### 7.2. Copper-64

Shortly after the pioneering work of Rossin et al., other radiometal PET isotopes such as ^64^Cu, ^89^Zr, ^68^Ga, or ^44^Sc, linked to different chelating agents, were used to radiolabel Tzs [73,79,83,84,87,88]. One of the first PET radionuclides used for Tz-radiolabeling was ^64^Cu by Lewis et al. in 2011. [^64^Cu]**7** was successfully used to label trastuzumab, a human epidermal growth factor receptor 2 (HER2) targeting vector [78]. These results inspired Lewis et al. to develop Tz agents for pretargeting in 2013 [79]. [^64^Cu]**2** was synthesized with a radiochemical purity (RCP) ≥ 99% and a specific activity (A_s_) of 8.9 ± 1.2 MBq/µg. This compound was evaluated in a SW1222 colorectal-cancer-xenograft-expressing A33 antigen in mice. The monoclonal antibody anti-A33, targeting A33, was modified with TCO (5 TCOs/mAb) [79]. Anti-huA33-TCO was used for pretargeted imaging and injected 24 h (100 µg, 0.66 nmol) prior the injection of [^64^Cu]**2** (1.2–1.4 mg, 10.2–12.0 MBq). Observed activity in the tumor 1 h p.i. was approx. 4.1% ID/g. Although images showed clear tumor uptake with good contrast, the authors concluded that the imaging agent is not ideal for clinical translation, as it accumulates in the intestine, which is not ideal for imaging colon cancer [79]. Consequently, [^64^Cu]**4** and [^64^Cu]**5** were developed. Both compounds were designed to possess a higher polarity, as it is known that increased polarity reduces intestinal uptake. This goal was achieved by incorporating a PEG linker into the lead, resulting in [^64^Cu]**4,** and by replacing the NOTA chelator with a more polar sarcophagus-based chelator (SarAr) linker, resulting in [^64^Cu]**5** [80]. [^64^Cu]**4** was radiolabeled with a radiochemical yield (RCY) of 78 ± 6% (d.c.), an RCP of >99%, and a molar activity (A_M_) of 11.9 ± 1.3 GBq/μmol, while [^64^Cu]**5** was radiolabeled with an RCY of 79 ± 7% (d.c.), an RCP of >99%, and an A_M_ of 11.5 ± 1.3 GBq/μmol. Both tracers were evaluated using the same cancer model and setup as described before. PET images of both candidates showed a better renal clearance and reduced uptake in the intestine, especially for [^64^Cu]Cu-SarAr-Tz ([^64^Cu]**5**). Tumor accumulation for [^64^Cu]**4** was determined to be 4.1 ± 0.3% ID/g 1 h and 3.9 ± 0.9% ID/g 24 h after administration**.** Tumor accumulation for [^64^Cu]**5** was determined to be 5.6 ± 0.7% ID/g 1 h and 7.4 ± 2.0 ID/g 24 h after administration. In comparison to conventional labeling of HuA33, pretargeting results in much better tumor-to-muscle ratio [80]. Similar pretargeting experiments were carried out using TCO-modified nanoparticles (TCO-NPs) as a targeting vector [81]. [^64^Cu]**8** was, in this case, used as an effector molecule. TCO-NPs (100 μg, 1 nmol TCO, 1 μg/μL) was injected into mice bearing U87 glioblastoma tumor xenograft 24 h prior to the injection of radiolabeled [^64^Cu]**8** (11 MBq/100 μL, 10 nmol Tz). High tumor-to-background ratios were observed, as was rapid clearance of the unreacted [^64^Cu]**8**. Tumor/liver-percent-injected dose per gram (% ID/g) was 16/17% ID/g 24 h p.i. for the pretargeted experiment, 3.5/24% ID/g for directly labeled NPs, and 1.6/3.7% ID/g for free [^64^Cu]**8** [81]. 

In light of the promising results of [^111^In]**1** and [^64^Cu]**4** with respect to pretargeting, a recent head-to-head comparison was carried out of both structures. [^111^In]**1** and [^64^Cu]**4** were tested in BALB/c nude mice bearing LS174T colon carcinoma xenografts and injected with CC49-TCO (100 µg, ~7 TCO/mAb, 3.9 nmol TCO) 72 h before the administration of the tracers. PET images of [^64^Cu]**4** showed a tumor uptake increasing from 3.2 ± 0.3% ID/g (2 h p.i.) to 7.7 ± 0.2% ID/g (22 h p.i.). These values were slightly lower than those obtained with [^111^In]**1**. However, the clearance profile of [^111^In]**1** was better at providing lower blood activity values. Despite minor differences, both tracers proved to be promising pretargeted agents (Figure 4) [75].

### 7.3. Gallium-68

The first pretargeted imaging study using a short-lived PET radionuclide, gallium-68 (68.4 min), was reported by Devaraj et al. in 2014 [82]. In this study, a highly reactive H-Tz was functionalized with diethylenetriaminepentaacetic acid (DTPA)-dextran polymer and labeled with gallium-68 ([^68^Ga]**3**). The polymeric dextran scaffold was chosen because of its well-established clinical safety record, its hydrophilicity, its easy availability in numerous molecular weights, its low cost, and its wide application as a radionuclide imaging agent [89,90,91,92]. As any reaction is dependent on the starting material’s concentration, this approach was thought to increase the in vivo click performance. Chelation to produce [^68^Ga]**3** succeeded using DTPA conjugated to dextran (8 mol per dextran) [93] in a 99% radiochemical conversion (RCC) [82]. For in vivo studies, the non-internalized A33 antigen, which is expressed in 95% of colon cancers, was chosen as a target [94,95,96]. Anti-huA33 antibody was TCO-modified (5.3 TCO/mAb) and used as a pretargeting vector. Mice implanted with LS174T xenografts were administrated pretargeting vector 24 h prior to the administration of [^68^Ga]**3** (1.85 MBq). PET imaging revealed a tumor-to-muscle ratio of 3.9 ± 1.8%ID/g 60 min p.i.; [^68^Ga]**3** was stable at least for 3 h. No significant differences were observed in the in vivo biodistribution compared to directly labeled ^68^Ga-DTPA-Dextran [82]. Therefore, it is not proven that [^68^Ga]**3** clicked to the targeting vector in vivo.

In 2014, the group around Aboagye developed another ^68^Ga-labeled Tz ([^68^Ga]**6**) for pretargeted PET imaging of cetuximab [85]. Cetuximab (C225, Erbitux), a chimeric human/murine IgG1 mAb that targets the epidermal growth factor receptor (EGFR/ErbB1), is indicated for the treatment of patients with colorectal and head and neck cancer. This mAb was approved by the FDA (Food and Drug Administration) in 2004 [97,98]. Cetuximab was TCO-modified, resulting in 17 TCOs moieties per mAb, and the Tz was radiolabeled with 75% RCY (end of synthesis (EOS)) and A_S_ of 40–55 GBq/µmol. The in vivo pretargeted strategy was carried out in A431-xenograft-bearing mice that were pretreated with TCO-modified cetuximab 3 or 23 h prior to the administration of [^68^Ga]**6** (~1.85 MBq). The results were in line with what was expected for pretargeted images. Accumulation in the tumor was lower in the 3 h treatment (0.86% ID/mL), while higher in background biodistribution in non-target tissues, compared to higher tumor uptake in the 23 h strategy (3.48% ID/mL), which had a 2.64 tumor/liver ratio. As negative controls, additional biodistribution studies of [^68^Ga]GaCl_3_ and [^68^Ga]**6** were performed. Tumor uptake of ^68^GaCl_3_ (from γ-counting) was observed to be 5% ID/g, compared to those of [^68^Ga]**6** and the pretargeting approach of 1.8 and 3.34% ID/mL, respectively. This was explained by the fact that ^68^GaCl_3_ has been reported to have high nonspecific uptake in some tumors [99]. The author proposed, moreover, that the high liver uptake observed for both the radiolabeled mAbs and pretargeted studies was a consequence of the high degree of TCO modification [85]. In summary, the results of this study are somewhat surprising, as other studies have shown that methyl-Tzs should not have fast enough rate constants for pretargeted imaging [86,100]. It is also surprising that the antibody could be modified without strong aggregation being observed. We believe that this study requires further experiments to be validated, as it is not clear if the observed tumor uptake stems in fact from dechelated gallium-68.

In a more recent study, a ^68^Ga-labeled analogue of [^111^In]**1** was developed by Edem et al. [84]. Labeling succeeded with a RCY of 48–78% with an RCP ≥ 95%. [^68^Ga]**11** was evaluated in two different pretargeting systems, namely the already-described CC49-TCO as well as an alendronate-TCO (Aln-TCO) platform. Aln is a bisphosphonate (BP) that has been used as a drug delivery vector to target bone tissue [101,102,103]. For example, technetium-99m methylenediphosphonate ([^99m^Tc]-MDP) is widely used for SPECT imaging of bone metastases, microfractures, and osteomyelitis. Therefore, Aln-TCO was developed to obtain access to a pretargeting system in naïve animals [104]. Biodistribution studies and PET imaging of [^68^Ga]**11** revealed target-specific uptake in bone (3.7% ID/g, knee) in Aln-TCO-pretreated mice as well as tumor-specific uptake (5.8% ID/g) in CC49-TCO-treated mice bearing LS174 xenografts. The study showed that [^68^Ga]**11** can be used as an alternative to [^111^In]**1** [84].

### 7.4. Scandium-44

In 2019, Edem et al. developed a ^44^Sc-Tz analogue of [^111^In]**1** [83]. The half-life of scandium-44 (3.97 h) is long enough for its production and transport while minimizing radiation dose. Scandium-44 also has a high positron branching ratio, resulting in high image quality [59,63]. Radiolabeling of [^44^Sc]Sc-DOTA-PEG_11_-Tz ([^44^Sc]**10**) resulted in a RCYs of 85–95% (n.d.c.) with an RCP > 99%, an A_M_ of 1 GBq/µmol (EOS), and an inherent stability of at least 24 h at 37 °C in saline and human serum albumin (HSA). The evaluation of [^44^Sc]**10** was performed using the Aln-TCO model described above in healthy Wistar rats. The opportunity to perform in vivo studies in healthy animals is advantageous, as no disease model is needed, which opens the possibility of testing pretargeted imaging in other species—in this case, rats. [^44^Sc]**10** was administered 1 h after Aln-TCO injection in a TCO:Tz ratio of 118:1. Accumulation could be observed in the shoulders, elbows, wrists, and spinal cord after 60 min. Activity uptake in the femur and humerus was 0.9 ± 0.3% ID/g, in kidneys 0.7 ± 0.2% ID/g, while in other tissues it was less than 0.1% ID/g. This accumulation was 30 times higher compared to untreated controls; elevated bone-to-blood (19.8 ± 0.4 femur, 18.9 ± 0.4 humerus) and bone-to-muscle (111.3 ± 0.4 femur, 106.0 ± 0.5 humerus) ratios were observed. This study demonstrated that [^44^Sc]**10** is a suitable imaging tool for pretargeting and that pretargeting can be carried out in larger animals than mice [83].

## 8. Non-Radiometal Radionuclides

Carbon-11 and fluorine-18 are radionuclides frequently used for PET imaging studies. Carbon-11 has advantages for test–retest studies, as it allows the performance of repeat scans in the same subject within the same day because of its short half-life (20.4 min). Fluorine-18 is the most relevant radionuclide in a clinical setting. It possesses near-optimal properties for this purpose. A half-life of approx. 110 min allows for a good distribution range of 200–300 km [49,64]. The radiation burden of fluorine-18 is typically acceptable and lower compared to other radionuclides such as carbon-11 or gallium-68. Positrons emitted by fluorine-18 also have relatively low energy, which ultimately results in good resolution using this nuclide in PET imaging studies. Other interesting applications of non-radiometal radionuclides include iodine isotopes as a SPECT, PET, and therapeutic radionuclide. 

### 8.1. Carbon-11

The first successful ^11^C-radiolabeled Tz was reported by Herth et al. in 2013 [105]. [^11^C]**12** was synthesized using [^11^C]MeI in a RCY of 33%, a RCP of 98%, and an A_M_ of 60 GBq/µmol. [^11^C]**12** was evaluated in mice and pigs. Pretargeting failed in the Aln-TCO pretargeting model. Interestingly, the tracers showed brain uptake in pigs within the first 5 min (SUV approx. 2.5–3.0) and a rapid washout. [^11^C]**12** was unfortunately not further evaluated, and it remains undetermined if [^11^C]**12** can ligate to targets beyond the BBB [106]. In 2016, another ^11^C-Tz was developed by Denk et al. [107]. [^11^C]**13** was successfully radiolabeled via ^11^C-methylation with a RCY of 52 ± 6% and RCP of 95% within 30 min [107]. Mesoporous silica nanoparticles (MSN) that were previously modified with TCO (116 µmol/g) or s-TCO (119 µmol/g) were used for the evaluation of [^11^C]**13** in BALB/c mice. These nanoparticles were administered and allowed to circulate for 5 min before [^11^C]**13** (15.4 ± 6.0 MBq was injected. In vivo binding was detected for MSN functionalized with s-TCO-NPs (1.5 SUV) and TCO-NPs (2.5 SUV). The lower activity concentration observed for MSN functionalized with s-TCO-NP compared to TCO-NPs could be explained by the fast isomerization of s-TCO in vivo [107].Recently, Herth et al. published a ^11^C-labeled Tz using Tz-stannane as precursors [108]. Labeling succeeded via copper-mediated ^11^C-carboxylation using [^11^C]CO_2_ with a RCY of 15 ± 5% (d.c.), an RCP of 99%, and an A_M_ of 11 ± 7 GBq/μmol. The labeling procedure is reported to be simple, reproducible, and fast. This strategy opens the possibility of radiolabeling Tzs for highly reactive structures suitable for in vivo pretargeting.

**Table 3:** Overview of ^11^C- and ^125^I-labeled Tzs.

NO.	Chemical Structure	Refs.	NO.	Chemical Structure	Refs.
12	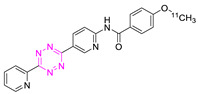	[105,106]	**14**	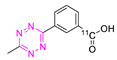	[108]
13	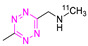	[107]	**15**	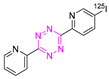	[109]

### 8.2. Iodine-125

A method to radiolabel bispyridyl-based Tz with iodine-125 was reported by Albu et al. (Table 3) [109]. Radiolabeling was carried out using a stannane precursor and “standard” iodination conditions. The reaction proceeded at room temperature and was completed in 15 min with an RCY of 80%. [^125^I]**15** was stable for 24 hours. The agent was not applied for pretargeted experiments, but for conventional labeling experiments of a TCO-anti-VEGFR2 conjugate, which was labeled with a RCY of 69% [109]. In order to use an iodine-labeled Tz derivative for pretargeted tumor imaging, the applied structure is required to be optimized with respect to its hydrophilicity. We have recently shown that only Tzs with a relatively low LogD_7.4_ (approx. −3 or below) are suitable for tumor pretargeting [86]. Additional other iodine isotopes more suitable for molecular imaging must be used—for example, iodine-124. Overall, the study by Albu et al. showed the feasibility of labeling even highly reactive Tz with iodine, but extensive modifications of this structure are needed for it to be suitable for pretargeted imaging.

### 8.3. Fluorine-18

Fluorine-18 is the most clinically employed radionuclide in PET [59,110,111]. This is due to its almost-ideal nuclear properties. Fluorine-18 has, for example, a half-life of approx. 110 min—long enough to produce and ship the radiotracer to other facilities. Fluorine-18 also possesses a good branching ratio for β+ of 96.7%, and its emitted positron has a relatively low average kinetic energy, resulting in a short positron range (2.4 mm max. range in water) [111,112]. These characteristics translate into high resolution, accessibility, and commerciality of ^18^F-based radiopharmaceuticals. Over the years, several efforts to directly label Tzs with ^18^F have been developed. The first unsuccessful attempt was made by Li et al. in 2010; only trace amounts of [^18^F]**16** could be detected (~1% RCC) [113]. Higher yields were not accessible due to the intrinsic instability against basic conditions, more precisely against “hard” nucleophiles. Their electron rich character makes them prone to nucleophilic attacks leading to the formation of undesired by-products. It is, therefore, not surprising that labeling strategies exploiting S_N_2 reactions, which require harsh conditions, resulted in low RCYs. Unfortunately, the instability against bases correlates to the Tz’s reactivity. Therefore, Tzs with the correct kinetic profile for pretargeting in vivo strategies cannot be labeled by applying “standard” labeling approaches. This issue was initially circumvented using indirect ^18^F-labeling strategies.

### 8.4. Indirect Labeling Strategies

As mentioned before, the harsh reaction conditions required for direct ^18^F-fluorination, such as high temperatures and the use of nucleophilic bases, disturb the labeling of highly reactive Tzs such as mono-substituted or bis-pyridyl scaffolds [114]. Indirect labeling methods can be used to solve this issue. In this approach, a synthon is first labeled under standard radiofluorination conditions and then conjugated to the Tz scaffold [18,86,115]. In this way, the Tz core is not affected by the harsh conditions required during the ^18^F-fluorination reaction.

The first indirect labeling was reported by Devaraj et al. in 2012. In this study, a polymer-modified ^18^F-Tz was developed and used in pretargeted PET. A dextran polymer was selected as a probe due to its low cost, high stability, and numerous in vivo applications [116,117,118]. The modification of the 10 kDa polymer consisted of a low-molecular-weight Tz fluorophore (MW 1.3 kDa) or polymer modified Tzs (PMT) (approximately 2 Tzs/polymer). ^18^F-fluorination of the Tz was carried out by indirect radiolabeling of (E)-5-(2-[^18^F]fluoro-ethoxy)cyclooct-1-ene, achieving an RCY of 89% (d.c.) and subsequent “click” with the Tzs on the polymer. For in vivo evaluation, mice bearing LS174T tumor xenografts were administered TCO-modified anti-A33 antibody (3 TCO/mAb) followed in a second step by [^18^F]**18** (30 µg, 5.55 MBq) after 24 h. PET/CT images 3 h p.i. of the Tz-dextran-based agent showed a clear difference between control mice and those that received TCO-anti-A33 mAbs 24 h [119]. This indicates that in vivo ligation has occurred in the tumor. However, biodistribution data of [^18^F]**18** in non-target tissues, as well as values for tumor uptake, were not reported. As such, it is not clear if uptake of [^18^F]**18** was driven by pretargeting or the enhanced permeability and retention (EPR) effect. However, these results inspired the same group to develop a similar approach with ^68^Ga-labeled Tz. The results of this work were reported earlier in this review and showed no difference in tumor accumulation between pretargeted mice and mice solely injected with the polymer-based pretargeting vector [82].

Isotopic-exchange (IE)-based silicon fluoride acceptor (SiFA) is an interesting strategy for indirect^18^F-labeling of Tzs. In the first step, the [^18^F]-SiFA-OH building block was prepared by simple isotope exchange (^18^F for ^19^F) with an RCY ≥ 97%. The latter was then reacted with 1,4-dichlorotetrazine to produce [^18^F]-SiFA-OTz ([^18^F]**19**) with an RCY of 92 ± 2% [120]. A total amount of 658.6–799.2 MBq of [^18^F]**19** could be synthesized with a typical A_M_ of 7.1 to 8.6 GBq/µmol in a total synthesis time of 25 min. This radiotracer was employed for the radiolabeling of other TCO-modified synthons, but not for pretargeting. This radiolabeling method proved to be simple and efficient; however, the influence of the lipophilic alkylated organosilicon moiety on the pharmacokinetics of compound [^18^F]**19** was not reported [120].

In 2015, Rashidian et al. established a new method for radiolabeling Tzs using commercially available ^18^F-fluorodeoxyglucose ([^18^F]FDG) [121]. The chemical equilibrium between the cyclical hemiacetal derivative and aldohexose in its linear aldehyde form led to the coupling of [^18^F]FDG on the aminooxy-functionalized Tz, yielding [^18^F]**20** with 90% RCC in 5–10 min. The ^18^F-fluorinated Tz was then used for in vitro ligation of TCO-modified anti-major histocompatibility complex (MHC) Class II single-domain antibodies with a 25% RCY (n.d.c.) to detect heterotopic pancreatic tumors in mice by PET imaging. Unfortunately, the biodistribution of [^18^F]**20** alone has never been reported [121]. A similar indirect strategy to radiolabel Tzs was developed by Keinänen et al. [122]. The focus of this work was to develop an optimal radiolabeling procedure for highly reactive Tz with favorable pharmacokinetics for in vivo pretargeting. ^18^F-fluorination was achieved by the aliphatic labeling (S_N_2) of 5-[^18^F]fluoro-5-deoxyribose ([^18^F]FDR) followed by oxime ether formation with the aminooxy-functionalized Tz precursor. [^18^F]**20** was radiolabeled in a total time of 2 h with an overall RCY of 50.5 ± 1.7% (d.c.), an RCP > 99%, and an A_M_ of 809 GBq/μmol. The stability of [^18^F]**20** was tested in mouse plasma (50% whole plasma in PBS) at 37 °C. The radiotracer proved to be stable for the first 2 h (90% RCP) and degrades by 50% after 6 h. In addition, an ex vivo biodistribution was performed in male BALB/c mice, with only 1% ID/g observed in bone after 60 min, demonstrating low levels of defluorination. The rapid clearance through the liver in the first hour and the rapid elimination in the urine confirmed that the low lipophilicity of the [^18^F]**20** promotes a favorable pharmacokinetic profile. These results, together with the fast reaction kinetics with TCOs in plasma (k_2_ = 4500 M^−1^ s^−1^), made [^18^F]**20** interesting for Tz in vivo application. Therefore, in a follow-up study, this compound was used as a pretargeting agent with healthy mice using TCO-modified microporous silicon nanoparticles(TCO-NPs) [123]. For this, TCO-NPs (0.2 mg, 1.42 nmol TCO) were injected 15 min. before i.v. administration of [^18^F]**20** (5.9 ± 0.5 MBq, Am = 22.2–40.2 GBq/µmol). The results revealed rapid in vivo binding after injection of the radiotracer, as the highest concentration of activity was observed in the liver and spleen after just 15 min, following the typical biodistribution pattern of NPs [124]. This corresponds to 9.8 ± 0.7% ID/g (60 min p.i.) in the spleen for animals pretreated with TCO-NPs and 1.0 ± 0.3% ID/g (60 min p.i.) for control animals (free [^18^F]**20** administration). Additionally, high activity concentration was observed in the lungs (13.9 ± 5.3% ID/g 30 min p.i.), most likely due to TCO-NP aggregation [107]. This study demonstrated for the first time that in vivo imaging with pretargeted PET has potential using TCO-modified nanoparticles and high A_M_.

A similar experiment was carried out by the same research group using two different TCO-modified mAbs, cetuximab and trastuzumab [125]. Prior to the pretargeted experiments, ^89^Zr-labeled mAbs were administered to tumor-bearing mice to understand their biodistribution. TCO-cetuximab (6 TCOs/mAbs) and TCO-trastuzumab (5 TCOs/mAbs) were used as pretargeting vectors and were injected 24, 48, or 72 h prior to the administration of [^18^F]**21**. A431- or BT-474-tumor-bearing mice, respectively, were used as a tumor model. [^18^F]**21** injection was performed in two different ways. Group A received ^18^F-Tz diluted with the same molar amount of unlabeled Tz. Group B was first administered unlabeled Tz (same amount as for group A) and 5 min later, [^18^F]**21** was administered. Overall, a total activity of 16–22 MBq of [^18^F]**21** was injected with the same A_M_. The highest tumor-to-blood ratio for TCO-cetuximab was observed after 72 h between antibody and [^18^F]**21** injections. There was no significant difference between the chosen time point for TCO-trastuzumab. PET images showed tumor uptake for both TCO-modified mAbs. Ex vivo biodistribution confirmed tumor uptake with 1.5 ± 0.1% ID/g and 3.7 ± 0.1% ID/g for TCO-trastuzumab and TCO-cetuximab, respectively. There were no significant differences in tumor uptake for mice in different groups (Group A and B). High amounts of radioactivity were observed in the blood for both mAbs, but to a much lesser degree with trastuzumab. 

In 2021 Steen et al. proposed a ^18^F labeling strategy based on copper-mediated click chemistry [86]. In this study, six different Tzs derivatized with alkene moieties were reacted with three types of ^18^F-labeled azides. The labeling was achieved with different RCYs (1–68%) based on the Tz scaffold and on the azide. The A_M_ obtained for the compounds varied considerably and was in the range of 5–230 GBq/μmol. The best compound of the series, [^18^F]**22,** was successfully labeled with an RCY of 11%, an A_M_ of 151 GBq/μmol, and an RCP ≥ 90% and was subsequently tested in vivo. Pretargeting PET imaging conducted with [^18^F]**22** in LS174T-xenografted tumor-bearing mice pretreated 72 h earlier with C49-TCO already demonstrated a mean tumor uptake of 1.7 ± 0.6% ID/g 1 h after the injection. The tracer showed moderate target-to-background ratios (5-fold) [86].

## 9. Direct ^18^F-Labeling Approaches

**Figure 5:**
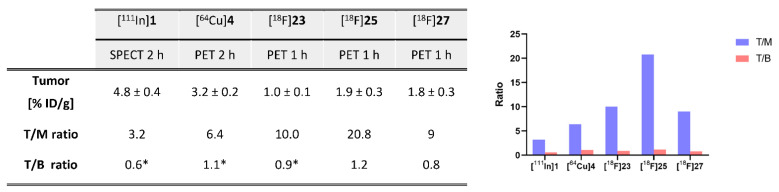
Image-derived tumor uptake (mean % ID/g), tumor-to-muscle ratio (T/M), and tumor-to-blood ratio (T/B) of “state-of-the-art” pretargeting imaging agents: [^64^Cu]**4** (PET 2 h p.i., *n* = 4), [^111^In]**1** (SPECT 2 h p.i., *n* = 4), [^18^F]**23** (PET 1 h p.i., *n* = 3), [^18^F]**25** (PET 1 h p.i., *n* = 4), and [^18^F]2**7** (PET 1 h p.i., *n* = 5). Tumor uptake and ratios of all Tz agents in nude BALB/c mice bearing subcutaneous LS174T tumor xenografts pretreated with CC49-TCO (100 mg). Data are shown as mean ± standard error of mean (SEM). * Image-derived uptake in heart from SPECT and PET images used as a surrogate for blood.

As described above, highly reactive mono- or bis-(hetero)aryl-substituted Tzs tend to decompose under the harsh conditions needed for standard nucleophilic ^18^F-fluorination (S_N_2 or S_N_Ar) approaches [113]. Therefore, the first labeling attempt failed [113]. In 2014, Denk et al. succeeded, for the first time, to radiolabel a Tz. However, the method could only be applied to low-reactive tetrazines. A methyl-Tz could be labeled with a RCY of 18% via ^18^F-aliphatic substitution (S_N_2). Biodistribution studies were carried out by administration of [^18^F]**17** in BALB/c mice followed by dynamic PET scanning and ex vivo quantification. The results showed favorable pharmacokinetics of the low-molecular-weight [^18^F]**17**: homogeneous biodistribution, brain uptake, rapid clearance, in vivo stability, and no bone accumulation [100]. However, the low reactivity hampered its use for pretargeting. In 2021, direct aromatic radiolabeling of Tzs succeeded. Cu-mediated fluorination using stannane precursors was the only possibility for labeling highly reactive Tzs [126]. Moderate-to-good RCYs (10–24%) were obtained for methyl-, phenyl-, and H-Tzs. Bispyridyl Tzs could not be radiolabeled due to the chelating effect of the pyridyl- scaffold. This methodology was used to develop a H-Tz for in vivo pretargeting purposes. [^18^F]**23** was produced with a RCY of 11 ± 3%, a molar activity of 134 ± 22 GBq/µmol, and an RCP > 99% in a total synthesis time of 90 min. Pretargeted PET imaging in xenograft-LS174T-bearing mice was carried out. Mice were administered CC49-TCO 72 h prior [^18^F]**23** injection. Imaging was carried out 1 h p.i. and resulted in a mean tumor uptake of 0.99 ± 0.14% ID/g. The tracer displayed rapid clearance and showed a good tumor-to-muscle ratio of 10. This ratio is significantly higher compared to “state-of-the-art” pretargeting imaging agents such as [^111^In]**1** and [^64^Cu]**4** (at same evaluation timepoints) (Figure 5). 

In order to extend the possibilities of labeling Tzs from aromatic to aliphatic approaches, Herth et al. directed their efforts toward identifying a method of radiolabeling base-sensitive compounds through aliphatic nucleophilic substitution (S_N_2) [127,128]. Optimizing preconditioning conditions for anion exchange cartridges and elution conditions for fluoride-18 trapped from them allowed the labeling of base-sensitive structures. With these findings in hand, the labeling of a highly base-sensitive H-Tz, [^18^F]**25**, succeeded with a RCY of approx. 20%. The use of non-nucleophilic bases and a careful selection of the solvent proved to be the key to achieving this goal. In two follow-up studies, these labeling conditions were applied to Tzs designed for in vivo pretargeting. Radiolabeling was performed under low basic conditions using [^18^F]Bu_4_NF/Bu_4_NOMsPO_4_^3−^ and nosylate precursors in a one-pot, two-step procedure for a total synthesis time of 90 min. [^18^F]**25**, based on H-Tz scaffold, was isolated with a RCY of 13 ± 2%, an RCP >98%, and a molar activity of 55 ± 5 GBq/μmol. Similarly, [^18^F]**27**, based on a bispyridyl scaffold, was radiolabeled with a RCY of 16%, an A_M_ of 57 GBq/µmol, and a high RCP >98%. The ability of both compounds to be used as pretargeting imaging agents was then evaluated. The compounds were tested in LS174T-tumor-xenografted mice. CC49-TCO was injected 72 h before the Tz imaging agents and mice were imaged 1 h p.i. Both tracers had a significantly higher tumor uptake (1.87 ± 0.31 for [^18^F]**25** and 1.81 ± 0.3% ID/g for [^18^F]**27**) than controls. The tumor uptake was clearly visible, and a high tumor-to-muscle (T/M) ratio was determined for both tracers (20 for [^18^F]**25** and 9 for [^18^F]**27**). A tumor-to-muscle ratio of 20—observed after 1 h—is thus far the highest ratio observed (Figure 5).

Recently, a new strategy to label Tzs with ^18^F was published. It is based on the sulfur fluoride exchange reaction (SuFEx). [^18^F]fluoride could be incorporated with an outstanding RCC of 100% using this method. The exchange is carried out at room temperature over 30 s without stirring. This method has not yet been applied to highly reactive Tzs and only succeeded using aliquot conditions [129]. Future studies must investigate if SuFEx can be scaled and if it is compatible for labeling highly reactive tetrazines.

**Table 4:** Overview of ^18^F-labeled Tzs from 2013–2021 (List is not exhaustive).

NO.	Chemical Structure	^18^F-Labeling Method	Refs.
**16**	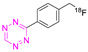	Direct aliphatic nucleophilic substitution (SN_2_)	[113]
**17**	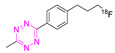	Direct aliphatic nucleophilic substitution (SN_2_)	[100]
**18**	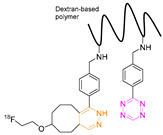	Indirect aliphatic nucleophilic substitution (SN_2_)	[119]
**19**	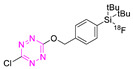	Indirect fluoride anion exchange	[120]
**20**	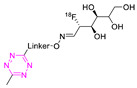	Indirect aliphatic nucleophilic substitution (SN_2_)	[121]
**21**	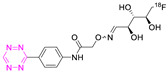	Indirect aliphatic nucleophilic substitution (SN_2_)	[122,123,125]
**22**	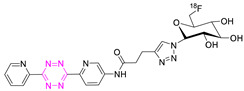	Indirect aliphatic nucleophilic substitution (SN_2_)	[86]
**23**	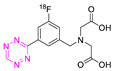	Direct aromatic labeling (oxidative fluorination)	[126]
**24**	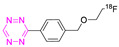	Aliphatic labeling (S_N_2)	[127]
**25**	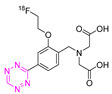	Aliphatic labeling (S_N_2)	[76]
**26**	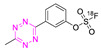	Fluoride anion exchange	[129]
**27**	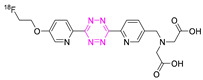	Aliphatic labeling (S_N_2)	[130]

## 10. Miscellaneous 

Aluminum [^18^F]fluoride labeling has been developed in order to label molecules with fluorine-18 in aqueous medium. In this method, the [^18^F][AlF]^2+^ cationic complex is formed and chelated using NOTA or NODA, for example. This is possible because of the strong interaction between aluminum and fluoride. Aluminum [^18^F]fluoride labeling is therefore a somewhat special case, as it can be seen more as a chelator approach than traditional ^18^F-labeling—with all advantages and disadvantages of such an approach. In general, aluminum [^18^F]fluoride labeling is in its infancy and is typically difficult to scale. However, many efforts have been reported and are made to improve labeling yields [131,132,133,134]. As such, aluminum [^18^F]fluoride labeling is a very interesting approach and was also applied to labeling Tzs. In 2015, Meyer et al. reported the first Tz labeling with aluminum-[^18^F]fluoride and NOTA precursor. ^18^F-fluorination of [^18^F]**28** was achieved with RCYs of 54–65% (d.c.), RCP >96%, and specific activities between 21.4 and 26.7 GBq/μmol. The stability of [^18^F]**28** was evaluated by injecting it into healthy athymic nude mice. Blood samples showed that 63% of [^18^F]**28** was still intact 4 h after injection. The activity concentration in bone was low (≤0.2% ID/g), demonstrating its stability. [^18^F]**28** was then evaluated in vivo using a TCO-bearing anti-CA19.9 mAb 5B1 (TCO-5B1) in a pancreatic xenograft mouse model. For pretargeting, TCO-5B1 (1.33 nmol) was administered 72 h before injection of [^18^F]**28** (1.33 nmol, 18–20 MBq). Biodistribution of the Tz tracer showed a tumor uptake of up to 6.4% ID/g (4 h post injection) [131]. Table 5 displays some examples of tetrazine labeled with aluminum [^18^F]fluoride.

## 11. Influence of Physicochemical Properties of Tz Derivatives on the Pharmacokinetic Profile

**Figure 6:**
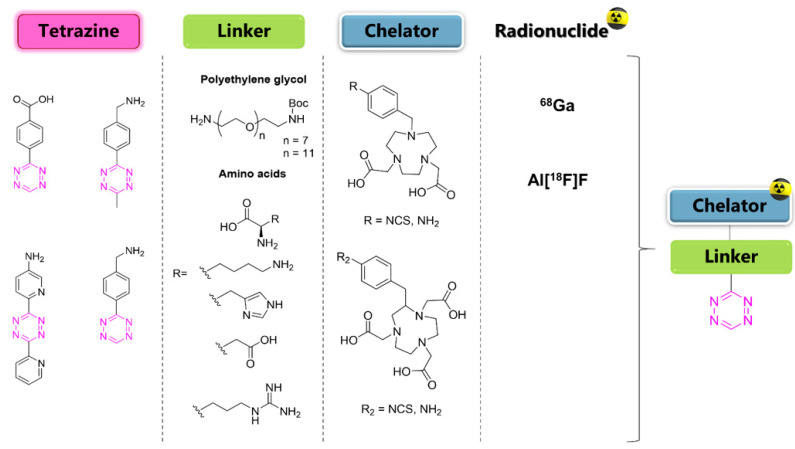
Modular approach for the synthesis of Tzs: Radioligands consisted of a Tz scaffold for in vivo click, a linker to alter the biodistribution, and a chelator to bind the radionuclide.

As shown above, the toolbox of radiolabeling reactions for Tzs has dramatically increased over the last two decades. Several methodologies are now available, unlocking the possibility of labeling Tz with different radionuclides. Progress in the field, however, is still hampered by the lack of rational design of Tz probes. Only a few studies exploring the relationship between physicochemical properties and pretargeting performance have been reported so far. Keinänen et al. were the first to suggest that “a low-lipophilicity, fast clearing tetrazine would be especially beneficial for pretargeted immunoimaging, resulting in substantially decreased radiation burden to non-target tissues in contrast to using a conventionally radiolabeled antibody construct” [122]. The in vivo data of [^18^F]**21** (Table 4), a glycosylated reactive tetrazine, demonstrated that this was the way to achieve a successful performance in vivo. The first systematic study on the physicochemical parameters needed for in vivo pretargeting was reported the next year by Zeglis et al. [45]. The objective of their work was to identify a suitable lead candidate for clinical development with high tumor uptake, but more importantly, to improve tumor-to-background ratios. A combinatorial approach with varying Tz scaffolds, linkers, and chelators was selected to explore several structures. Chelation of Al[^18^F]F was chosen to radiolabel the Tzs since the procedure was already developed and also allowed a switch to ^68^Ga as an alternative radionuclide if desired. Accordingly, a total of 25 new Tz-imaging agents were designed and synthesized in order to identify which physicochemical properties are associated with optimal pharmacokinetic in vivo (Figure 6). Three Tz scaffolds (-methyl, -H, and -byspiridyl Ts) with different kinetics were selected. Several linkers attached to the Tz-structure, consisting of polyethylene glycol (PEG_7_ or PEG_11_), amino acids (AA) [AA = lysine, histidine, aspartate, and arginine], or a combination of both, were explored. Finally, two distinct bifunctional chelators, 1,4,7-triazacyclononane-1,4-diacetic acid (NODA) and 1,4,7-triazacyclononane-1,4,7-triacetic acid (NOTA), were explored. Each compound obtained in this way had different molecular parameters and properties, such as overall molecular net charge, in vivo stability, distribution coefficient, lipophilicity (clogD_7.4_), and plasma half-life (PHL) (Figure 6). All ^18^F- and ^68^Ga-labeled Tz derivatives were successfully labeled with a high RCY >55% for ^18^F-Tzs and >83% for ^68^Ga-Tzs, with A_M_ >19 GBq/µmol and high RCP >95%. In vivo evaluation was performed in the same way described in previous studies: injecting TCO-modified mAb 5B1 (TCO-5B1) 72 h before Tz administration. The results showed few trends. The overall molecular charge of the radiotracers had an essential influence on the behavior in vivo and on the clearance profile. High net charge reduced lipophilicity; shortened plasma half-life (PHL) inducing renal clearance. The compound with neutral net charge showed a higher absorption in the liver and intestines.

This trend was observed for compounds [^18^F]**29** and [^68^Ga]**9**, with a charge of 0 and +1, respectively. The use of AA residues as linkers in the structures reduced circulation times in the bloodstream and influenced renal and hepatic clearance. On the other hand, the introduction of lysines in the linker strongly decreased the PHL tracer to less than 6 min, while the substitution of lysine by other AAs such as arginine influenced renal accumulation. Consequently, substitution of lysine with arginine or histidine reduced renal uptake 4-fold. The plasma half-life of the radiotracers affected the concentration of tumor activity, suggesting that a half-life longer than 10 min is needed to achieve good tumor accumulation. In the pretargeted evaluation, the main compounds [^18^F]**29** and [^68^Ga]**9**, containing PEG_11_ linkers and molecular charges of 0 and +1 showed PHL of 15.1 min and 17.1 min, respectively. Tumor accumulation was 7.6 ± 1.8% ID/g and 6.8 ± 1.4% ID/g 2 h p.i. for [^18^F]**29** and [^68^Ga]**9**, respectively, and good tumor-to-background ratios were observed. Biodistribution confirmed that the neutral charge of [^18^F]**29** promoted longer PHLs, and thus better tumor absorption, but also higher background levels due to slower excretion through the intestines. The study by Meyer at al. shed some light on the parameters required to obtain Tzs with good performance for pretargeting [45]. However, the number of compounds and structural similarities between Tz scaffolds were still a limiting factor, and some questions regarding the reaction kinetics necessary to achieve in vivo click were unanswered. 

**Figure 7:**
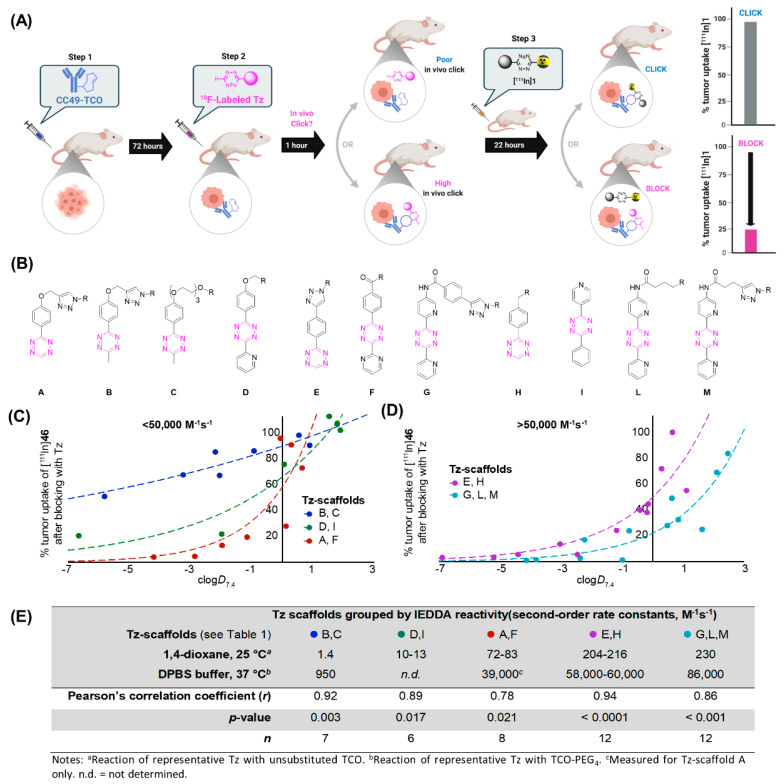
Results from the blocking assay. The blocking effect of non-radiolabeled Tz was calculated as the change in tumor uptake of [^111^In]**1** 22 h p.i. (**A**) General scheme of the blocking assay. First, tumor-bearing mice were administrated with CC49-TCO, 72 h before injection of a nonradioactive Tz. After 1 h, [^111^In]**1** was injected, and ex vivo biodistribution was performed 22 h p.i. to determine the blocking effect of the non-radiolabeled ^19^F-Tz. The uptake was normalized to a group of animals (control) in which no blocking was performed. (**B**) Structural Tz scaffolds. (**C**,**D**) Correlation of clogD_7.4_ and blocking effect for Tz derivatives with alike IEDDA reactivity. (**E**) Statistical analysis of the correlation between blocking and tumor uptake for the different groups of Tz. Pearson’s correlation coefficient (r) describes the fit between the clogD_7.4_ and blocking effect. Modified with permission from ACS. Copyright © 2021, American Chemical Society.

In 2021, Steen et al. carried out a similar approach aiming to identify the key parameters needed to obtain an optimum Tz-based radiotracer [86]. The crucial step of this study was the development of a pretargeted blocking assay that allowed for the investigation of the in vivo fate of the structurally diverse libraries of several unlabeled Tzs and their capability to reach and react with TCO-modified mAbs in tumor-bearing mice. This assay is a very powerful tool since it omits the time-consuming development of radiolabeled Tzs for every ligand to be tested. It is based on the ability of Tzs to block the binding of pretargeted imaging agent [^111^In]**1** to the pretargeting vector CC49-TCO (administered 72 h prior) in tumor-bearing mice. The tumorblocking effect of the unlabeled Tz derivatives is afterwards determined by ex vivo biodistribution and normalized to the binding of [^111^In]**1** without any blocking. The setup is displayed in Figure 7. A structurally diverse library of 45 Tz derivatives was tested. The compounds had different properties such as calculated TPSAs values or clogD_7.4_ values. Moreover, the Tz scaffolds considered in the study included mono- and disubstituted methyl-, phenyl-, 2-pyrimidyl-, and 2-pyridyl-substituted Tz derivatives with a broad range of second-order rate constants for the reaction with TCO (Figure 7). The results obtained are shown in Figure 7. The “blocking effect” strongly correlated with clogD_7.4_ and the reactivity of the selected Tzs. Low-reactivity Tzs, such as scaffolds B and C, are not able to achieve a full block even at lower clogD_7.4_, proving that this type of molecule is not suitable for in vivo use. On the other hand, it was demonstrated that the more reactive the Tz is, the less polarity is needed to achieve a full block (e.g., scaffold G, L, M). The authors suggested that high rate constants (>50,000 M^−1^ s^−1^) for the reaction with TCO and low clogD_7.4_ values (below -3) are needed for successful pretargeting. This hypothesis was confirmed by labeling the most promising compounds in the blocking assay and testing them in vivo, including compound [^18^F]**27** [86].

## 12. Future Perspective

Tz ligation has emerged as one of the most promising pretargeted tools for in vivo applications. This is because of its outstanding reaction kinetics, selectivity, and yield. This click reaction enables the efficient labeling of nanomedicines in live cells and even in vivo. Therefore, the radiolabeling of Tzs has come into focus. Several methods have been developed within the last decade, spanning from chelator to nucleophilic substitution approaches. However, relatively few approaches have been described for radiolabeling Tzs with therapeutic nuclides [25,104,135,136,137,138,139]. We believe new therapeutic and theranostic Tzs will emerge for pretargeting strategies within the next years. More importantly, in late 2020, the first clinical phase I trial based on tetrazine ligation was initiated (NCT04106492) [24]. In this pilot study, a TCO-modified targeting vector can be activated in vivo after reacting with a selected Tz to release a chemotherapeutic. We believe that radiolabeled Tzs with suitable properties for in vivo pretargeting might became a useful tool to quantify the release of the chemotherapeutic and/or to achieve an additional therapeutic effect. In general, tetrazine ligation and its unique reaction properties are still unfolding; its potential to be used for diagnostic or therapeutic applications can potentially revolutionize theranostic applications in nuclear medicine.

## Figures and Tables

**Table 1:** Physical properties of common radionuclides for molecular imaging [59,60,61,62,63].

Modality	Isotope	Half-Life	Branching Ratio(β^+^) (%)	Maximum Positron Range in Water (mm)	Gamma-Photon Energy (keV)
**PET**	Carbon-11 (^11^C)	20.4 min	99	4.5	-
	Gallium-68 (^68^Ga)	68.4 min	88	10.3	-
	Fluorine-18 (^18^F)	109.8 min	97	2.3	-
	Copper-64 (^64^Cu)	12.7 h	17.6	2.9	-
	Arsenic-72 (^72^As)	26.0 h	88	18.2	-
	Zirconium-89 (^89^Zr)	78.4 h	22.7	4.2	-
	Scandium-44 (^44^Sc)	4.04 h	94.3	2.3	
	Iodine-124 (^124^I)	100.2 h	22.8	11.7	-
**SPECT**	Iodine-123 (^123^I)	13.3 h	-	-	159
	Indium-111 (^111^In)	67.3 h	-	-	171 and 245
	Technetium-99m (^99m^Tc)	6.01 h	-	-	140

**Table 5:** List of aluminum [^18^F]fluoride labeled Tz.

NO.	Chemical Structure	^18^F-Labeling Method	Refs.
**28**	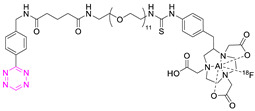	Direct labeling using Al [^18^F]F-chelating ligand	[131]
**29**	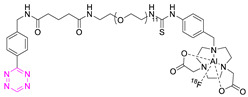	Direct labeling using Al [^18^F]F-chelating ligand	[45]

## Data Availability

Not applicable.
